# Treatment rationale in nutcracker syndrome with concurrent pelvic congestion syndrome

**DOI:** 10.1186/s42155-025-00527-0

**Published:** 2025-02-14

**Authors:** Dominik A. Steffen, Arash Najafi, Georgios Festas, Christoph A. Binkert

**Affiliations:** 1 Department of Radiology and Nuclear Medicine, Cantonal Hospital Winterthur, Brauerstrasse 15, Winterthur, CH-8401 Switzerland; 2 2nd Department of Radiology, Attikon University General Hospital, Athens, Greece; 3 Medical Radiological Institute Zurich, Zurich, Switzerland

**Keywords:** Nutcracker syndrome, Stenting, Pelvic congestion syndrome, Gonadal vein embolization

## Abstract

The optimal management strategy of nutcracker syndrome is debated, especially in the setting of concurrent pelvic congestion syndrome. In this article, we describe our treatment algorithm as illustrated by four different case scenarios. In our experience, renocaval pressure gradients are often inconclusive, but evaluation of the left renal vein waveform as well as a “test PTA” with evidence of a waist in the balloon can be helpful in unmasking a relevant stenosis. We consider nutcracker syndrome not to be a contraindication for ovarian vein embolization. Decision for simultaneous or sequential stenting should be based on angiographic findings and clinical course.

## Background

Nutcracker syndrome (NCS) refers to a symptomatic compression of the left renal vein (LRV) as it passes either between the superior mesenteric artery (SMA) and the aorta (“classic” or “anterior nutcracker”) or posteriorly between the aorta and the spine as an anatomical variant (“posterior nutcracker”). Patients typically exhibit left flank pain, hematuria and proteinuria and may develop secondary gonadal vein insufficiency as a result of collateral formation. The term “nutcracker syndrome” should be limited to symptomatic patients since a physiological tapering of the left renal vein in its aortomesenteric portion can be frequently observed in asymptomatic and, in particular, slender patients. This imaging finding is commonly referred to as “nutcracker phenomenon” [1–3]. NCS poses a diagnostic and therapeutic challenge due to the non-specific nature of symptoms, a lack of standardized diagnostic criteria and uncertainties regarding optimal treatment. In this article, we describe our therapeutic approach for patients with suspected NCS exemplified by four illustrative cases, with a focus on concurrent pelvic congestion syndrome (PCS).

### Cases

**Fig. 1:**
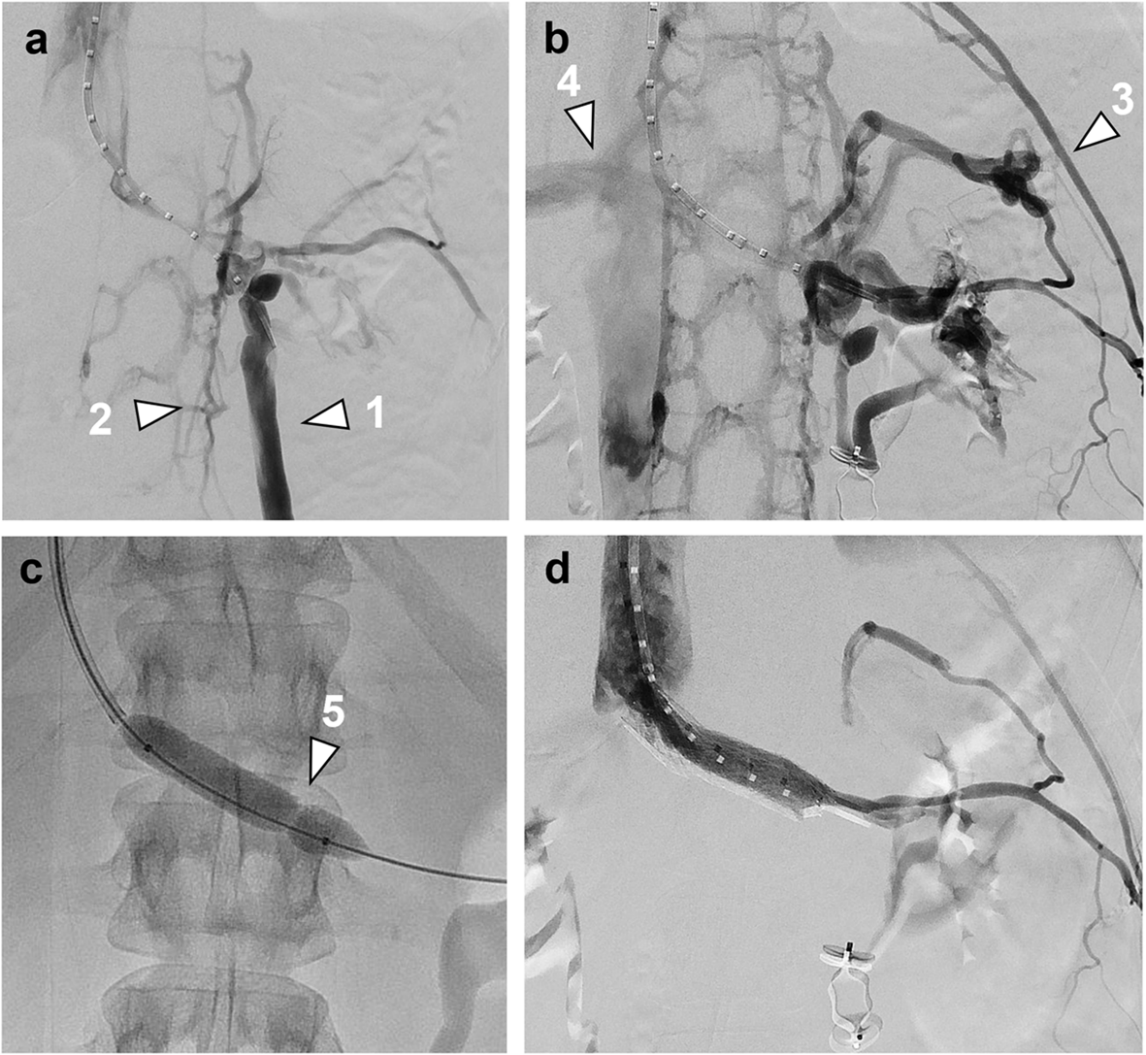
**a** Digital subtraction venography depicting a severely refluxing left ovarian vein (1) and paravertebral venous collaterals (2) in patient 1. The aortomesenteric portion of the LRV is not opacified. **b** After embolization of the left ovarian vein, paravertebral and renosplenic (3) collateral flow is noticeably increased with opacification of the portal vein (4). **c** On inflation of a 10 mm PTA balloon, a significant waist can be seen (5). **d** After implantation of a 12 mm self-expandable stent, collateral flow is significantly reduced

**Fig. 2:**
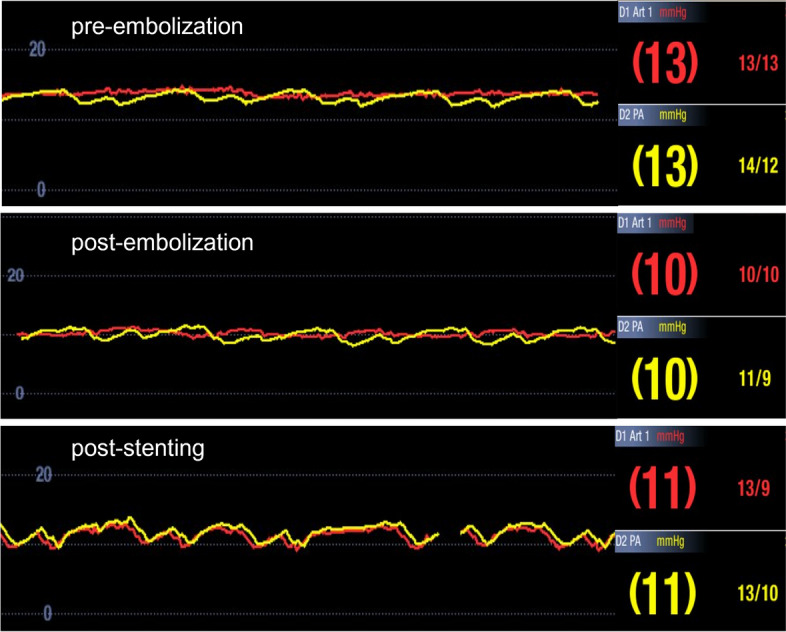
Pressure curves in the LRV (red) and the IVC (yellow) show no renocaval pressure gradient at baseline (top), after embolization of the left ovarian vein (middle) and after stenting of the LRV (bottom). Note the restored cardiac modulation of the LRV waveform after stenting

Patient 1 was a 20-year-old nulligravidous female referred to the IR clinic on suspicion of PCS. She complained of strictly left-sided pelvic and flank pain. Urine analysis was normal. MR angiography was unable to visualize the aortomesenteric segment of the LRV. The left ovarian vein was dilated up to 11 mm and the aortomesenteric angle was 14°. On venography, pronounced paravertebral and renosplenic collaterals and a severely dilated and refluxing left ovarian vein were observed (Fig. 1a). Simultaneous pressure measurements in the inferior vena cava (IVC) and the LRV showed no renocaval pressure gradient (0 mmHg) but a flattened LRV waveform without the fluctuations normally seen due to atrial contraction (Fig. 2). After embolization of the left ovarian vein, collateral flow was noticeably increased, especially the renosplenic pathway with opacification of the portal vein on delayed images (Fig. 1b). Notably, there was no increase of the renocaval pressure gradient (0 mmHg) (Fig. 2). Next, balloon angioplasty of the aortomesenteric segment of the LRV was performed, where a marked waist in the PTA balloon (10 mm) was observed (Fig. 1c). A 12 mm self-expandable stent (EverFlex, Medtronic, Dublin, Ireland) was subsequently deployed, after which venography showed brisk antegrade outflow in the LRV with substantially reduced collateral flow (Fig. 1d) and the pressure waveform in the LRV demonstrated restored cardiac modulation (Fig. 2). At 24 months follow-up, the patient remained symptom-free and had no stent-derived complications.

**Fig. 3:**
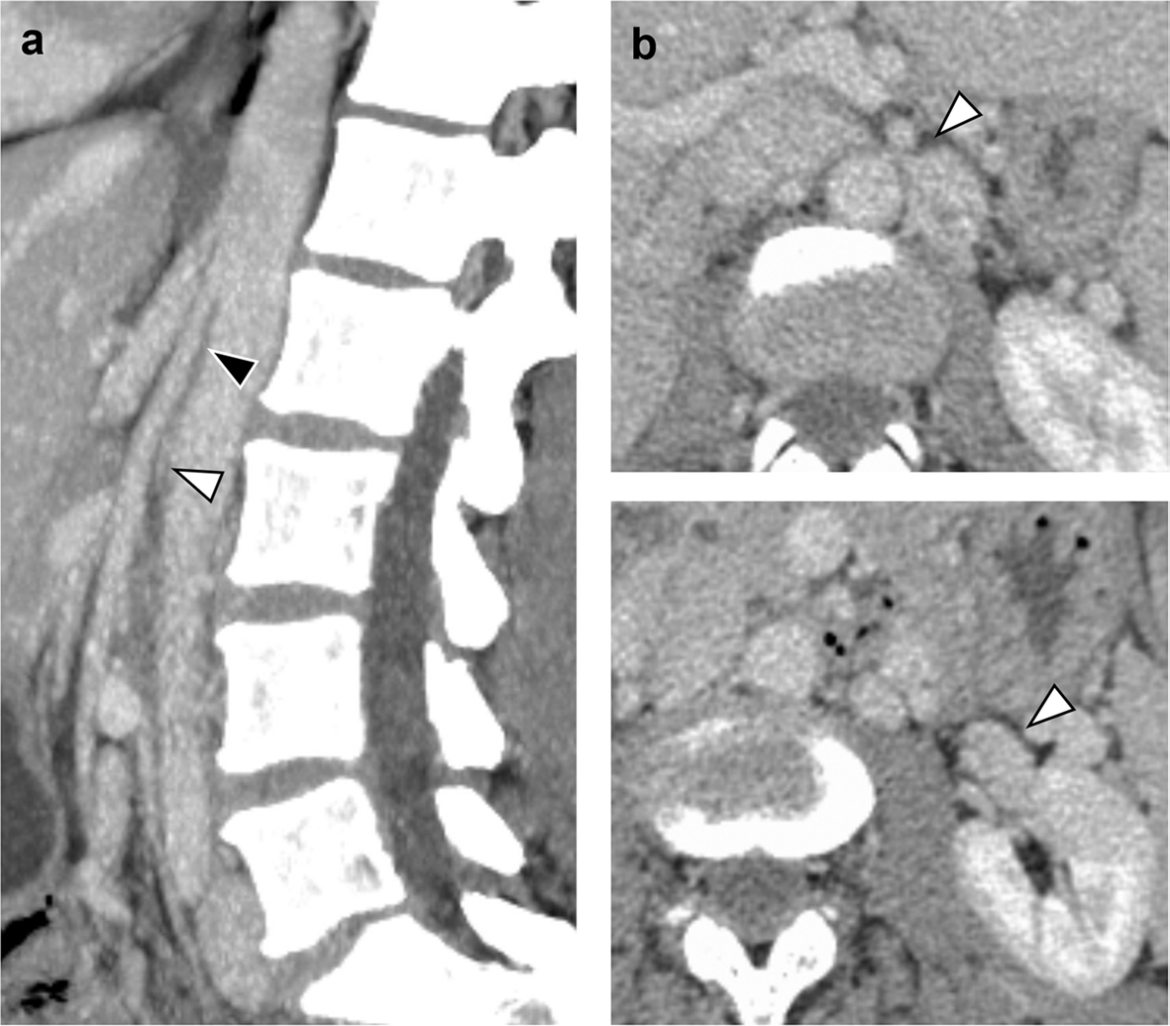
Typical nutcracker anatomy in patient 2. **a** Sagittal reformatted CT shows a near-parallel course of the proximal SMA (black arrowhead) and the aorta. Note the slit-like compression of the LRV (white arrowhead) **b** Axial slices depict a focal narrowing of the aortomesenteric portion of the LRV with „beak sign “ and a hilar-to-aortomesenteric diameter ratio of > 5

Patient 2 (f, 47y, two children) presented with left-dominant pelvic and flank pain exacerbated by lying on her left side and accompanied by microhematuria. CT and MR angiography showed an aortomesenteric angle of 7° (Fig. 3a), a typical “beak sign” with a hilar-to-aortomesenteric LRV diameter ratio of 5 (Fig. 3b) and a severely dilated (12 mm), refluxing left ovarian vein. Catheterization of the LRV proved difficult with considerable resistance felt at the LRV ostium. Because pressure gradient measurements showed no change in gradient (1 mmHg) before and after embolization of the left ovarian vein, no angioplasty or stenting of the LRV was performed. However, the patient returned to the IR clinic within a few days after the procedure reporting near-resolution of the pelvic pain but a sharp increase in the left flank pain. Repeat venography on suspicion of decompensated NCS revealed renosplenic collaterals while resistance was once again felt upon catheterization of the LRV. Balloon angioplasty with 12 mm followed by implantation of a 16 mm self-expandable stent (Zilver Vena, Cook, Bloomington IN, USA) was performed. The patient’s symptoms were quickly alleviated with no recurrence or stent-related complications at 38 months.

**Fig. 4:**
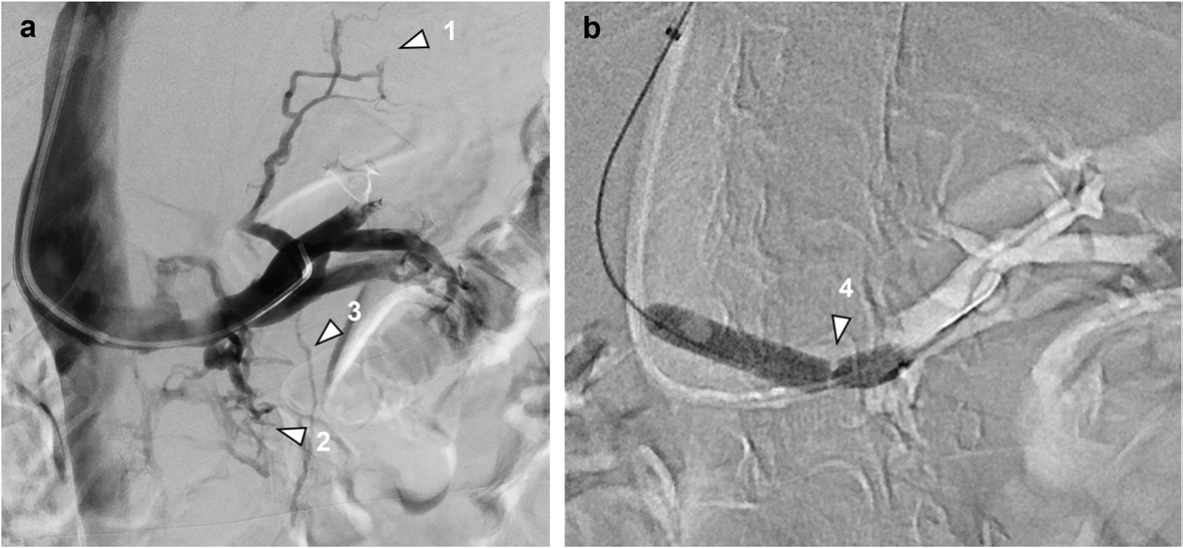
**a** Digital subtraction venography in patient 3 shows renosplenic (1) and paravertebral (2) collaterals, but only a diminutive left ovarian vein (3). **b** A significant waist (4) is observed on dilatation of a 10 mm PTA balloon

Patient 3 (f, 43y, 2 children) presented with long-standing, strictly left-sided flank pain and microhematuria alongside symptoms suggestive of PCS. She had a history of hysterectomy due to endometriosis and multiple episodes of urolithiasis. CT angiography revealed a retroaortic course of the LRV with marked narrowing in this segment, suggestive of posterior nutcracker syndrome (hilar-to-aortomesenteric ratio 2.6). The ovarian veins and the periuterine venous plexus were notably not dilated. Like in case 3, resistance was felt upon catheterization of the LRV. Venography showed pronounced retroperitoneal collaterals but only a small left ovarian vein (Fig. 4a). A renocaval pressure gradient of 2 mmHg was measured. Like in case 1, a marked balloon waist was observed while dilating the retroaortic segment of the LRV with 10 mm (Fig. 4b). After implantation of a 14 mm stent (Zilver Vena, Cook, Bloomington IN, USA) complete regression of the retroperitoneal collaterals was achieved.

**Fig. 5:**
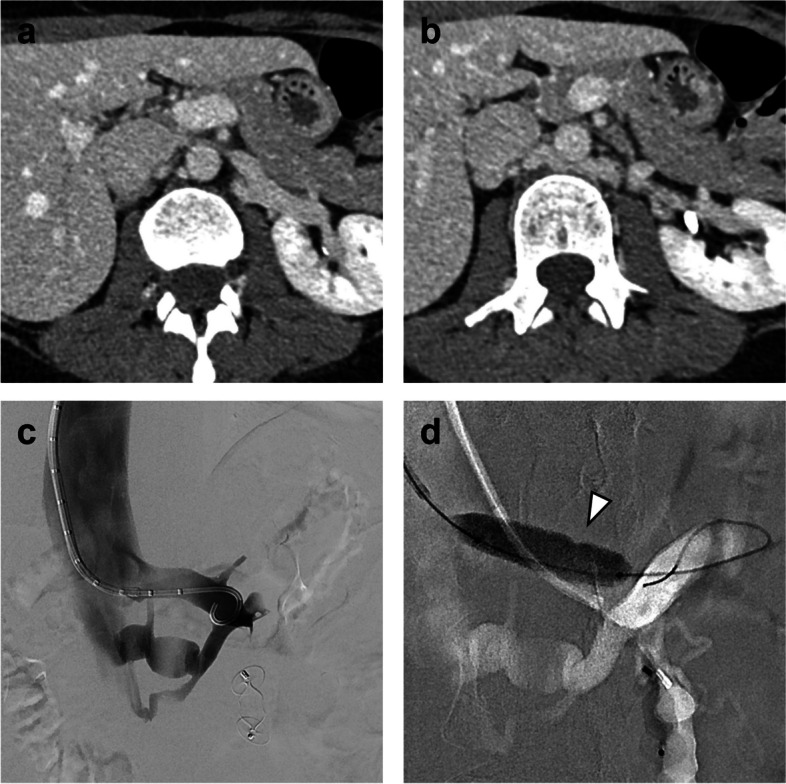
Circumaortic course of the LRV with separate pre- and retroaortic branches (**a** and **b**, respectively) in patient 4. **c** After embolization of the left ovarian vein, no collaterals are seen on venography. The more cranial preaortic and the more inferior retroaortic branches are clearly depicted. **d** Only a minimal waist is observed in the 12 mm PTA balloon (arrowhead)

Patient 4 (f, 51y) was referred on suspicion of PCS. Additionally, she experienced left sided flank pain and intermittent microhematuria. On CT angiography, the left ovarian vein was dilated up to 9 mm feeding into a dilated periuterine venous plexus. Further, the patient had a circumaortic LRV, i.e. a branching pattern with one vein branch passing between the aorta and the SMA and another branch passing posteriorly to the aorta. While the anterior branch had a morphology suggestive of nutcracker phenomenon with a hilar-to-aortomesenteric ratio of 6.1 and an aortomesenteric angle of 28°, the posterior branch demonstrated only a mild tapering of the retroaortic portion (Fig. 5a and b). Catheterization of the ventral branch was straightforward, and venography showed no collateral vessels apart from the refluxing left ovarian vein. After embolization of the left ovarian vein, repeat contrast injection confirmed rapid outflow through the LRV branches without any other collaterals (Fig. 5c). A “test PTA” of the preaortic LRV branch with a 12 mm balloon showed only a minimal waist (Fig. 5d). We therefore decided against stenting the preaortic LRV branch. The patient experienced an increase in left flank pain during the first week after the intervention with subsequent complete remission of symptoms and no recurrence at 17 months.

### Clinical presentation and non-invasive imaging

Overall, nutcracker syndrome is a rare entity and remains a diagnosis of exclusion after more common conditions such as urinary tract infections, urolithiasis, nephritis and endometriosis have been ruled out [4, 5]. As such, diagnosis of nutcracker syndrome is challenging and often delayed. Renal venous hypertension typically manifests as left-sided flank pain, hematuria or proteinuria. Secondary gonadal vein insufficiency may lead to pelvic congestion syndrome or left sided varicocele in females and males, respectively. Since the symptoms must be carefully correlated with imaging findings, interventional radiology plays a crucial role in both diagnosis and therapy.

Cross-sectional imaging readily depicts the acute narrowing of the aortomesenteric portion of the LRV, commonly termed “beak sign” on axial slices. The hilar-to-aortomesenteric LRV diameter ratio has been reported to have a specificity of 89–100% for NCS with cutoffs between 4.9–5.0 [6, 7]. Further, an aortomesenteric angle < 35–41° on sagittal reformatted images is nearly always present in NCS patients [1, 4–6]. This is commonly encountered in slender young patients with only little retroperitoneal fat, and it has been argued that this is the reason why NCS may be alleviated by weight gain in adolescents [1, 2, 5, 8]. In our experience, collaterals are usually not readily apparent on CT and MRI, with the exception of the gonadal veins.

### Invasive imaging and endovascular treatment

Catheter-based renal venography with renocaval pressure gradient measurement is the gold standard for the diagnosis of nutcracker syndrome [5, 9]. Renal hypertension is commonly defined as a renocaval pressure gradient of ≥ 3 mmHg [1, 7, 10]. Yet, as most healthy individuals exhibit no renocaval pressure gradient at all (≤ 1 mmHg) [11], some authors classify 1–3 mmHg as borderline [6, 12]. Furthermore, as seen in all cases presented in this article, paravertebral, gonadal and in extreme cases even renosplenic collaterals (Figs. 1b and 4a) may decompress the LRV stenosis, resulting in normal or borderline pressures (“compensated nutcracker syndrome”) [5, 6, 9, 12]. As case 4 shows, retroperitoneal collaterals and accessory LRV branches may also completely alleviate symptoms, while in other patients, such as cases 1 and 3, symptoms may persist despite extensive collateral formation (“decompensated nutcracker syndrome”). Therefore, pressure gradients are often inconclusive or even misleading. However, morphological evaluation of the LRV waveform can be helpful in such cases, with a loss of normal cardiac modulation indicating an obstruction of LRV outflow (Fig. 2).

On angiography, reduced opacification of the central portion of the LRV and presence of collaterals are the main visual indicators for nutcracker phenomenon. Sometimes, resistance can be felt upon catheterization of the LRV, indicative of a stenosis [13]. We found that test inflation of an appropriately sized PTA balloon (8–12 mm) with evidence of a waist may also be helpful in unmasking a *structural* LRV stenosis and differentiating this from a *dynamic* vascular compression (Figs. 1 and 4).

**Fig. 6:**
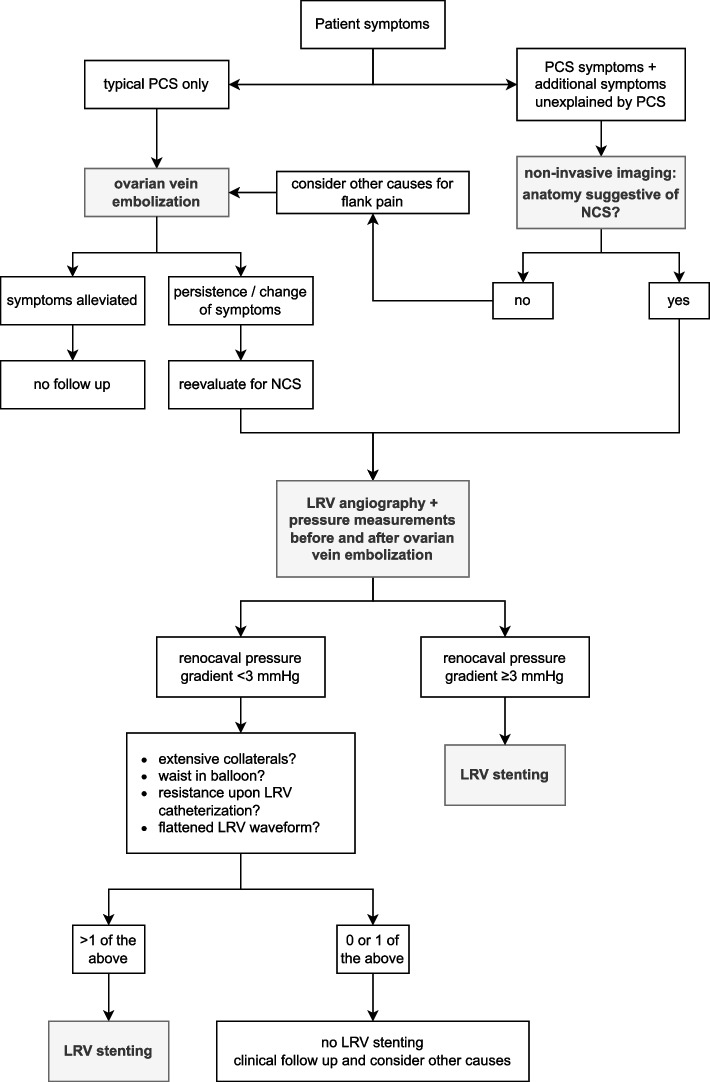
Proposed treatment algorithm used in our institution for patients with PCS and suspected NCS

The therapeutic approach used in our institution for patients with suspicion of NCS or PCS, which is based on the above rationale and builds on previously published algorithms [2, 9, 14, 15], is presented in Fig. 6. In patients with *typical* PCS, we do not routinely perform venography of the LRV prior to and after embolization of the left ovarian vein to rule out concurrent NCS. However, as exemplified by the cases presented in this article, suspicion of NCS should be raised if the patient exhibits symptoms not explained by PCS alone (i.e. renal congestion symptoms), if their symptoms change or deteriorate after gonadal vein embolization or if the patient does not match the typical patient population for PCS (e.g. a nulligravidous female). At first, we were hesitant to embolize the left gonadal vein in PCS patients with suspected concurrent NCS, as closing off one of the main collaterals may aggravate congestion in the LRV, exemplified by case 2. However, even in this patient, there was no increase in the renocaval pressure gradient immediately after left ovarian vein embolization. Therefore, gonadal vein embolization is unlikely to cause acute organ-threatening congestion. In borderline cases, it may be prudent to wait and see whether symptoms are spontaneously resolved, since two-staged embolization and stenting can still be done if necessary [15–17].

## Conclusion

Apart from the clinical presentation and symptom severity, the primary considerations when deciding whether to stent the LRV should be the extent of collaterals on angiography, resistance during LRV catheterization and the presence of a significant waist in an appropriately sized PTA balloon. As demonstrated above, renocaval pressure gradients may be misleading in cases of compensated NCS, but a flattened LRV waveform may indicate a relevant stenosis. In our view, NCS is not an absolute contraindication for gonadal vein embolization and does not necessarily require simultaneous LRV stenting in every case.

## Data Availability

The datasets analyzed during the current study are available from the corresponding author on reasonable request.

## Abbreviations

LRV: Left renal vein
NCS: Nutcracker syndrome
PCS: Pelvic congestion syndrome
SMA: Superior mesenteric artery
IVC: Inferior vena cava
